# Peripheral Neuromodulation and Opioid Sparing Strategies for Mitigating Perioperative Pain in the Stabilization and Hardware Removal of Complex Trimalleolar Fractures: A Case Report

**DOI:** 10.1002/ccr3.70713

**Published:** 2025-07-28

**Authors:** Bi Mo, Sandra Sacks, Jerry Markar

**Affiliations:** ^1^ Division of Pain Medicine, Department of Anesthesiology and Perioperative Medicine, David Geffen School of Medicine University of California, Los Angeles Los Angeles California USA; ^2^ Division of Hematology‐Oncology, Department of Internal Medicine, David Geffen School of Medicine University of California, Los Angeles Los Angeles California USA

**Keywords:** complex orthopedic fractures, hardware removal, opioid‐sparing strategies, perioperative pain management, peripheral neuromodulation, trimalleolar fractures

## Abstract

Individualized opioid‐free perioperative management, combining dual continuous peripheral nerve catheters targeting the popliteal sciatic and adductor canal nerves, non‐opioid pharmacologic agents, and adjunctive neuromodulation, offers robust multimodal analgesia. This approach supports both ORIF and hardware removal in complex trimalleolar fractures while preserving function, reducing opioid‐related risks, and promoting faster recovery.

## 1 Introduction

Complex trimalleolar fractures comprise approximately 7%–15% of all ankle fractures and are associated with the poorest functional outcomes among ankle fracture subtypes [1, 2, 3]. Annual incidence is estimated at 20–40 per 100,000 individuals, with a higher prevalence in osteopenic/osteoporotic geriatric females, often resulting from low‐energy supination–external rotation mechanisms [1, 3, 4].

Systemic opioids remain the conventional cornerstone of perioperative analgesia; however, their use in ankle fracture surgery, including trimalleolar fractures, has been statistically associated with an increased risk of new persistent opioid use (NPOU), defined as continued opioid consumption between 90 and 180 days following the index procedure [5]. Notably, adverse sequelae include opioid‐induced hyperalgesia, endocrine suppression, delayed fracture healing, and large societal costs [6, 7, 8, 9] while the long‐term benefits of opioids remain elusive [5, 7, 8, 9]. Given these well‐documented risks and the limited evidence supporting long‐term opioid efficacy, there is an urgent need to prioritize safer, evidence‐based alternatives for perioperative pain control. Accordingly, continuous peripheral nerve blockade (CPNB), multimodal non‐opioid protocols, and adjunctive neuromodulation have emerged as effective strategies that align with current AAOS guidelines for postoperative pain management [10, 11].

We present a case involving a 38‐year‐old athlete who sustained a right ankle trimalleolar fracture‐dislocation and who achieved opioid‐free analgesia during both the delayed open reduction and internal fixation (ORIF) and the elective hardware removal 12 months later. This was accomplished through a dual‐catheter CPNB protocol, complemented by non‐opioid pharmacologic adjuvants and adjunctive neuromodulation strategies.

## 2 Case Presentation

### 2.1 Part 1. Initial Stabilization via ORIF

#### 2.1.1 History and Examination

A 38‐year‐old male sustained a fall injury with immediate pain and inability to bear weight on the right ankle while playing indoor racket sports. Examination in the Emergency Department revealed a closed deformity with tense soft tissue edema, preserved perfusion, intact neurologic function, and pain with active and passive ankle motion.

#### 2.1.2 Investigations and Acute Management

**FIGURE 1:**
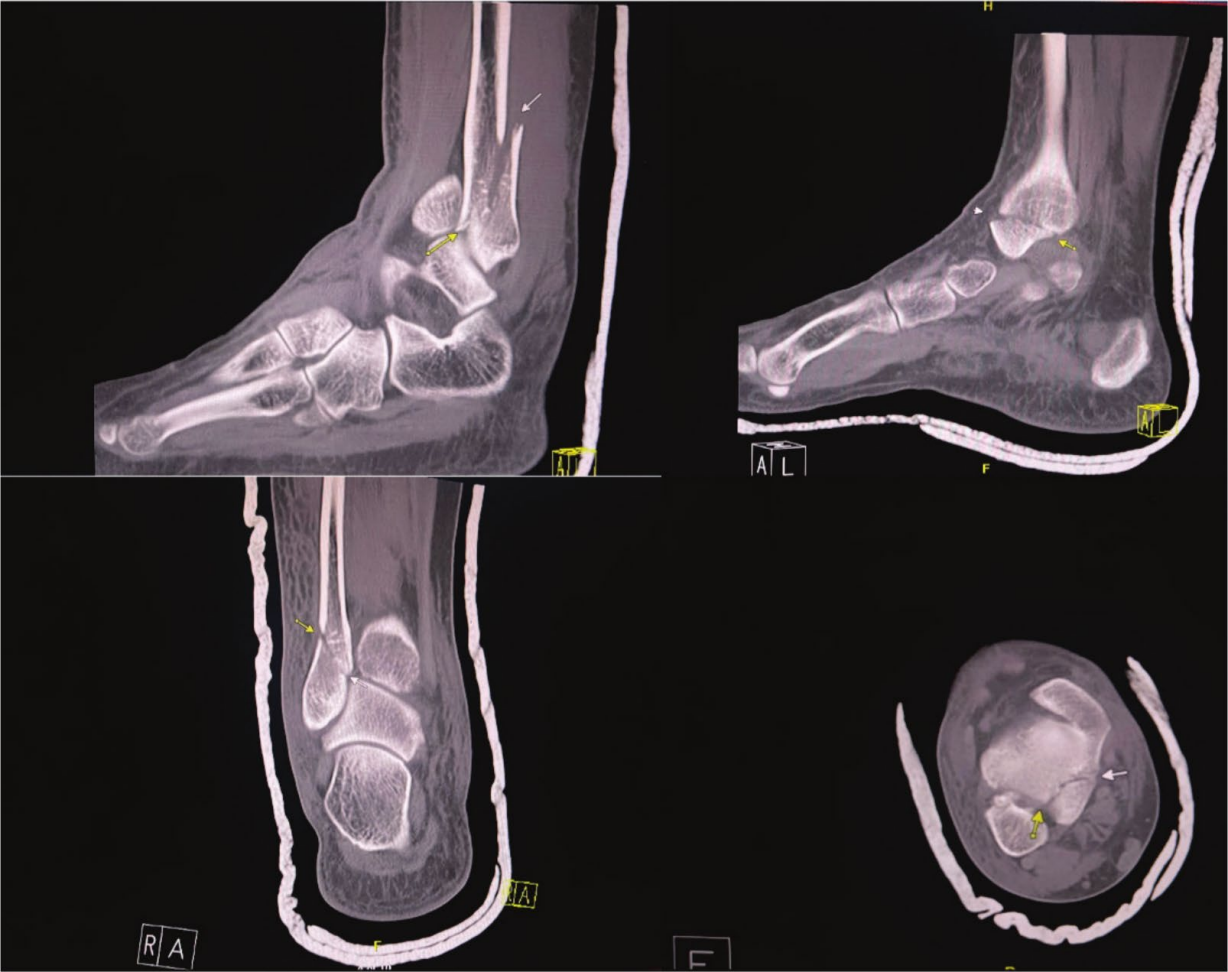
CT scan of the right lower extremity showing a trimalleolar fracture affecting the medial and posterior tibia and lateral fibula (yellow arrows). Image taken post‐manual reduction of ankle joint dislocation and plaster cast application in the emergency department.

**FIGURE 2:**
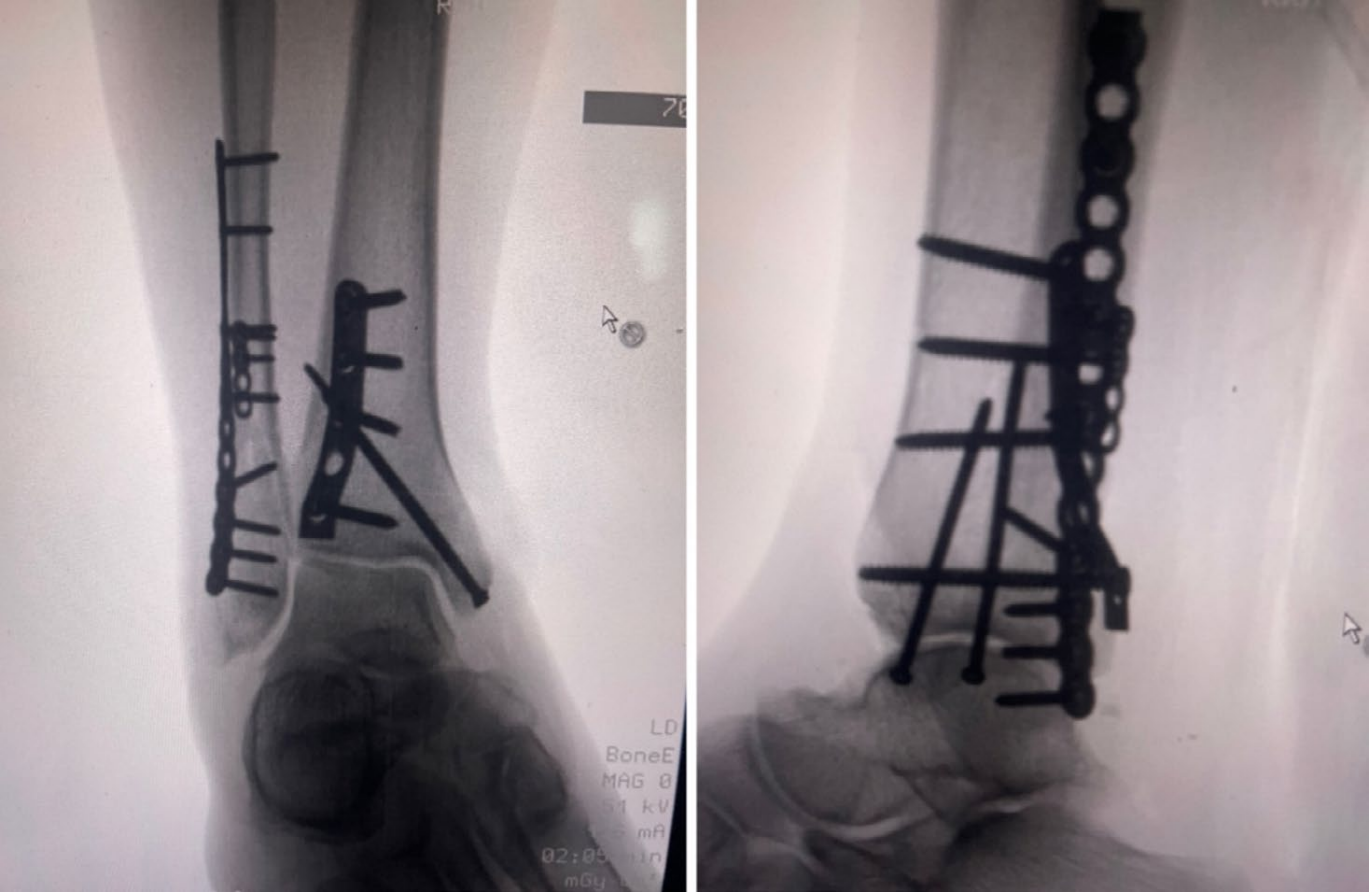
Intraoperative fluoroscopy post‐initial ORIF stabilization. Anteroposterior and lateral views show extensive hardware placement and successful reduction of trimalleolar fractures.

CT confirmed a right ankle trimalleolar fracture with a comminuted distal fibular fracture at the syndesmotic level, displaced medial and posterior malleolus fragments, and lateral talar subluxation (Figure 1). The dislocation was reduced under a talocrural hematoma block and immobilized in a below‐knee cast. The patient used RICE and non‐opioid analgesics while awaiting reduction of acute soft tissue. Sixteen days later, ORIF via a posterolateral approach employed a one‐third tubular plate for the posterior fragment, a reconstruction plate and flex plate for the fibula, and lag screws for the medial malleolus (Figure 2). Dual popliteal sciatic and adductor canal catheters with 0.1% ropivacaine at 2–4 mL h^−1^ for 5 days provided opioid‐free analgesia postoperatively.

#### 2.1.3 Postoperative Course

By Day 12, the incision was well‐healed and pain averaged 3/10 on the visual analog scale (VAS), with peaks to 5/10 when dependent; analgesics included acetaminophen, ibuprofen, and home transcutaneous electrical nerve stimulation (TENS). The patient resumed desk duties during Week 1, began weight‐bearing at Week 6, ambulated in a CAM boot by Week 7, and wore normal footwear by Week 8 with independent stair use. At 3 months postoperatively, residual dorsiflexion and plantarflexion deficits persisted; pain averaged 1–3/10, managed with topical diclofenac, acetaminophen, and twice‐weekly physiotherapy.

### 2.2 Part 2. Hardware Removal

#### 2.2.1 History and Examination

Twelve months post‐ORIF, the patient reported swelling and activity‐limiting pain over the fibular and medial plates; examination showed dorsiflexion 5°, plantarflexion 20°, and implant tenderness.

#### 2.2.2 Investigations and Procedure

Radiographs confirmed union without failure. Elective hardware removal was completed utilizing the same dual‐catheter CPNB protocol (0.1% ropivacaine at 2–4 mL h^−1^ for 5 days), with no systemic postoperative opioid use; adjuncts included acetaminophen, ibuprofen, TENS, and RICE.

#### 2.2.3 Recovery and Follow‐Up

Weight bearing as tolerated was immediate. By Day 3, the patient returned to work with VAS 1–3/10; rehabilitation began in Week 3 with significant swelling reduction (pre‐op VAS 3–4 vs. post‐op VAS 0–1/10). Scar management included triamcinolone and laser therapy, resulting in full activities of daily living, improved range of motion, and negligible edema.

## 3 Discussion

### 3.1 Clinical Challenge of Trimalleolar Fractures

Trimalleolar fractures represent a convergence of pain‐generating mechanisms, including multicolumn articular disruption, capsular stripping, marked peri‐injury edema, and extensive activation of periosteal nociceptors [1, 2, 3, 4]. This subcategory of ankle fractures is unstable, with potential for ligamentous and/or vascular injury requiring ORIF for stabilization to maximize recovery and optimize functional outcomes [1, 3]. Nevertheless, peri‐injury soft tissue swelling can be significant and pose challenges for immediate surgical intervention. The timing of surgical intervention is therefore highly variable among orthopedic specialists, and it can take up to 2–3 weeks prior to surgical stabilization [1]. Delayed ORIF, often required to allow adequate resolution of soft tissue swelling, may inadvertently extend the inflammatory phase and exacerbate perioperative pain [1, 2, 3]. The posterolateral surgical approach, commonly employed for posterior malleolar fixation, adds to soft tissue trauma and increases the risk of postoperative scarring [1, 2, 3, 12].

Even following fracture union, retained hardware along the fibula or posterior tibia may chronically irritate adjacent peroneal tendons and neurovascular structures, contributing to plate‐related pain reported in up to 20% of cases and frequently leading to consideration for hardware removal [12, 13, 14]. Despite its clinical relevance, large‐scale systematic studies evaluating the prevalence and functional impact of hardware‐related pain following ORIF are lacking, and thresholds for explantation remain inconsistently defined among surgeons [12, 13]. Hardware‐related pain remains a leading indication for implant removal, though other clinical factors, including deep late infection, metal hypersensitivity or toxicity, tumorigenic potential, implant migration, mechanical failure, and secondary fractures at plate termini, also contribute to the decision‐making process, as outlined by Böstman et al. [14]. Importantly, sufficient time must be allowed for bony union to consolidate before explantation is considered. As such, the timing of hardware removal for pain or related complications varies widely across orthopedic practices, with most guidelines recommending deferral of elective removal until at least 12 months post‐ORIF [12, 13, 14].

### 3.2 Opioid Related Morbidity: Magnitude and Mechanisms

Large administrative datasets report that NPOU develops in approximately 2.6% to 18.5% of previously opioid‐naïve orthopedic patients, with risk influenced by factors such as the initial prescription size and potency, as well as preoperative use of other controlled substances [15, 16, 17]. Patients undergoing lower extremity fracture surgery are among the highest‐risk groups. Beyond the risk of dependence, chronic opioid use is associated with a doubling of all‐cause mortality, increased susceptibility to infection, and impaired fracture healing, likely mediated through opioid‐induced immunosuppression, hypothalamic–pituitary–adrenal axis dysregulation, and direct suppression of osteoblast activity [7, 9, 15, 16, 17]. Furthermore, opioid‐induced hyperalgesia, mediated via NMDA receptor activation and spinal glial sensitization, may amplify nociceptive input and perpetuate chronic pain syndromes [7, 17]. Together, opioid use disorder and overdose have contributed to an estimated annual healthcare burden exceeding US $35 billion as of 2017 [9]. In response, contemporary clinical guidelines and expert consensus strongly support the adoption of multimodal, opioid‐sparing perioperative strategies to mitigate the incidence and consequences of NPOU [11, 18].

### 3.3 Dual Catheter CPNB: Anatomical Coverage and Advantages

The popliteal sciatic catheter provides reliable sensory blockade of the tibial and common peroneal nerves distal to their bifurcation, offering critical analgesia for the posterolateral surgical corridor and periosteal nociception along the fibula and posterior tibia. Supplementation with an adductor canal catheter effectively captures saphenous and articular obturator nerve fibers, territories often incompletely anesthetized by the sciatic block alone, thereby extending coverage to the anterior and medial ankle, including the medial malleolar fixation site, while preserving quadriceps motor function [19, 20]. Continuous infusion of low concentration ropivacaine/bupivacaine at 2–4 mL h^−1^ through an indwelling catheter with a pressure‐activated pump device maintained consistent analgesia for 3–5 days, significantly surpassing the 3–6 h duration of single‐shot spinal anesthesia (SA), which frequently results in rebound pain and opioid rescue therapy in up to 72% of cases [20]. Meta‐analyses have demonstrated that CPNB reduces the incidence of perioperative pain, hypotension, utilization of vasoactive pharmacologic agents, urinary retention [18, 21]. Nevertheless, CPNB itself is inadequate for intraoperative pain reduction (needs to be combined with general anesthesia or SA intraoperatively) or to prevent tourniquet‐induced pain [21, 22].

Although potential complications of CPNB include catheter migration (7%–12%), neurovascular injury (0.5%–1%), systemic local anesthetic toxicity, and infection rates below 0.5%, these risks are significantly mitigated through modern practices such as ultrasound‐guided placement, improved catheter devices, cyanoacrylate mesh fixation, and dilute local anesthetic solutions [19, 20, 21, 22, 23]. Cost‐utility analyses have shown that ambulatory care with CPNB is a safe and effective alternative to inpatient care following complex foot and ankle surgeries, reducing total costs by more than 50% while also emphasizing the need for prospective, non‐retrospective study designs to better account for patient‐specific factors and optimize implementation [24].

### 3.4 Tourniquet Induced Hyperalgesia and Mitigation

Thigh tourniquet application is employed in below‐the‐knee orthopedic procedures to reduce intraoperative blood loss and maintain a bloodless surgical field [25, 26]. However, this technique carries notable risks, including potential neurologic injury, and significantly increases perioperative pain through mechanisms involving ischemia–reperfusion injury [25, 26, 27]. This process is postulated through sensitization of dorsal horn neurons and activates spinal glial pathways, contributing to systemic hyperalgesia and prolonged postoperative pain, but the precise mechanism remains elusive [22, 25, 26]. The use of individualized limb occlusion pressure combined with elastic cuffs, rather than traditional fixed‐pressure protocols and conventional cuffs, may offer advantages by maintaining effective hemostasis while potentially reducing neural injury and tourniquet‐associated pain. Nevertheless, further high‐quality studies are needed to better characterize the safety and efficacy of this approach [25, 26, 27]. These concerns have prompted interest in analgesic techniques that can attenuate tourniquet‐induced pain and mitigate its systemic effects. In this context, Dabir et al. conducted a randomized clinical trial demonstrating that CPNB using a dual‐catheter approach, targeting the femoral nerve and lateral femoral cutaneous nerve in addition to the popliteal sciatic nerve, was effective in suppressing intraoperative tourniquet pain and postoperative rebound nociception during short‐ to medium‐duration foot and ankle surgeries, particularly in elderly patients with significant cardiopulmonary or anatomical challenges [22].

### 3.5 Hardware Removal: Second Hit Phenomenon

Revision procedures, such as hardware removal, particularly when undertaken through prior surgical corridors, can reactivate localized inflammatory pathways and increase the risk of transecting scar‐encased neurovascular structures [12, 13]. These factors offer a mechanistic rationale for the elevated postoperative pain scores and delayed functional recovery frequently reported following implant explantation [12, 13, 14]. Despite these anatomical and physiological challenges, the utilization of a dual CPNB regimen, targeting the popliteal sciatic and adductor canal nerves, provided effective analgesia in this patient, with immediate postoperative VAS scores maintained at 1–3/10. This reproducibility highlights the reliability and efficacy of this opioid‐free strategy across both primary and revision settings. Although residual stiffness persisted postoperatively, likely attributable to peri‐incisional fibrosis and soft tissue adhesion, substantial functional gains were achieved through a multidisciplinary rehabilitation protocol comprising targeted physiotherapy, intralesional triamcinolone injections, and fractional laser therapy for scar remodeling.

### 3.6 Risk of Acute Compartment Syndrome in Lower Leg Trauma

The application of regional anesthesia in orthopedic trauma, particularly in cases involving tibial fractures, has historically been approached with caution due to longstanding concerns regarding its potential to obscure signs of acute compartment syndrome (ACS), which has an estimated incidence of 3.1 (range: 1–7.3) per 100,000 persons [28]. While breakthrough pain remains a cardinal feature of ACS, its development is typically associated with intra‐compartmental pressures exceeding 30 mmHg, or when compartment pressure rises within 10–30 mmHg of the patient's diastolic blood pressure, thresholds indicating impaired perfusion and relative ischemia of the affected limb [29]. Dense regional blocks, particularly those utilizing high‐concentration or long‐acting local anesthetics, have been speculated to delay the recognition of ACS, potentially resulting in catastrophic consequences [28, 29].

However, accumulating evidence indicates that when dilute local anesthetics are used judiciously within a CPNB framework, sufficient sensory feedback can be preserved to allow recognition of evolving ACS symptoms. Importantly, no causal relationship has been established between regional anesthesia and delayed diagnosis of ACS when appropriate clinical vigilance is maintained [28, 29]. Contemporary best practices emphasize low‐concentration infusions, motor‐sparing techniques, and structured postoperative monitoring to balance analgesic effectiveness with diagnostic safety [28, 29].

In the presented case, the dual‐catheter CPNB approach facilitated opioid‐free analgesia while preserving the patient's awareness of compartment‐related symptoms and enabling safe, functional mobility. With appropriate patient selection, thorough preoperative counseling, and multidisciplinary coordination, including predefined escalation protocols for suspected ACS, regional anesthesia can be safely and effectively integrated into the perioperative care of complex lower‐extremity fractures. These findings support a paradigm shift from avoidance to thoughtful implementation of regional techniques in orthopedic trauma, anchored by vigilant monitoring and individualized care planning.

### 3.7 Neuromodulation Adjuncts

Integrating non‐pharmacologic neuromodulation techniques alongside CPNB may further enhance analgesic efficacy while preserving motor function integrity [30, 31]. TENS achieves analgesia by activating large‐diameter afferent fibers (A‐α and A‐β), which inhibit nociceptive transmission at the level of the spinal dorsal horn [30, 31, 32]. High‐frequency TENS (20–100 Hz) exerts its effects primarily through segmental gating mechanisms and activation of descending inhibitory serotonergic pathways, leading to suppression of C‐fiber activity [32]. In contrast, low‐frequency TENS (1–10 Hz), which operates via endogenous opioid release, demonstrates reduced efficacy in opioid‐tolerant patients [32].

Evidence from meta‐analyses across orthopedic, gynecologic, and thoracic surgical cohorts supports the utility of TENS, showing significant reductions in both postoperative pain scores and opioid consumption during the first 48 h compared to sham stimulation [30, 32]. High‐frequency TENS is particularly advantageous due to its lack of motor fatigue and ease of application during early mobilization and rehabilitation [30, 32].

Extending beyond TENS, percutaneous peripheral nerve stimulation (PNS) offers a promising modality for sustained, targeted analgesia. In a multicenter, randomized, double‐blinded, sham‐controlled pilot trial, Ilfeld et al. [31] demonstrated the feasibility and efficacy of ultrasound‐guided PNS using a helical micro‐lead implanted approximately 2 cm from the target nerve, typically the sciatic nerve for ankle procedures or the femoral nerve for knee surgeries. Electrical stimulation at 100 Hz was titrated from zero, with programmable modulation of current amplitude (0–30 mA) and pulse duration (10–133 μs), producing sub‐threshold paresthesia‐based analgesia throughout the perioperative period [31].

The study reported that PNS significantly reduced postoperative pain and opioid consumption during the first postoperative week in patients undergoing moderate‐to‐severe ambulatory orthopedic surgery. In a subset of patients, these analgesic effects persisted beyond lead removal on postoperative Day 14. Participants also experienced reduced pain interference with emotional and physical function throughout the 2‐week stimulation period and immediately afterward; however, these benefits diminished at 1‐ and 4‐month follow‐up assessments [31].

As part of a tiered, multimodal analgesic strategy, PNS may serve as a transitional bridge between dense catheter‐based blockade during the acute inflammatory phase and patient‐controlled neuromodulation during subacute recovery. This staged integration of peripheral neuromodulation aligns with the core principles of Enhanced Recovery After Surgery (ERAS) protocols and holds promise for further reducing reliance on systemic opioids in postoperative orthopedic care [18, 30, 31, 32].

### 3.8 Functional and Economic Outcomes

Although the operated ankle remained immobilized following initial fixation, the motor‐sparing profile of the adductor canal and popliteal CPNBs preserved proximal lower‐extremity function, specifically quadriceps and hamstring strength [19, 21]. This preservation of motor integrity enabled safe transfers, stable seated positioning, and independent home mobility following index ORIF. Following hardware explantation, weight‐bearing as tolerated was permitted immediately, allowing the patient to resume on‐site clinical operative responsibilities by postoperative Day 3. This advantage allowed both procedures to be completed on an outpatient basis. This rapid functional reintegration, coupled with avoidance of inpatient admission and elimination of opioid‐related adverse effects, meets previously established cost‐effectiveness thresholds for continuous peripheral nerve catheter protocols and aligns with broader principles of value‐based perioperative care [18, 24].

While the single‐case design inherently limits generalizability, the reproducibility of outcomes across both the index surgery and subsequent hardware removal enhances the external validity of this approach. Future multicenter randomized controlled trials (RCTs) are needed to evaluate the broader applicability of individualized opioid‐sparing pathways. These studies should stratify participants by fracture complexity and surgical approach and incorporate validated outcome instruments such as the Patient‐Reported Outcomes Measurement Information System Pain Interference (PROMIS‐PI), the Foot and Ankle Ability Measure–Activities of Daily Living subscale (FAAM‐ADL), opioid disposition at 90 days, and comprehensive metrics of healthcare utilization and cost‐effectiveness. The integration of these endpoints will be essential for developing standardized, evidence‐based guidelines that support the safe and scalable implementation of non‐opioid perioperative protocols in complex orthopedic trauma.

## 4 Conclusion

This case illustrates the feasibility and efficacy of an opioid‐sparing perioperative strategy in the management of complex trimalleolar ankle fractures, utilizing dual CPNB, non‐opioid pharmacologic agents, and adjunctive neuromodulation. The reproducibility of this regimen across both the initial ORIF and subsequent hardware removal highlights its clinical robustness. Pain was consistently well controlled without compromising motor function, allowing for early mobilization, independent recovery, and timely return to work, all without reliance on systemic opioids.

A critical factor in the success of this approach is thoughtful patient selection. Candidates who express strong motivation for opioid avoidance, possess adequate insight and functional independence, and can reliably report symptoms, even under regional anesthesia, are most likely to benefit from this strategy. In this case, the patient was highly engaged, motivated, and medically optimized, reinforcing the importance of tailoring opioid‐sparing protocols to the individual rather than applying them universally.

Importantly, this case also addresses long‐standing concerns regarding regional anesthesia in lower extremity trauma, including the potential to obscure signs of ACS. With dilute local anesthetic concentrations and structured postoperative monitoring, sensory feedback was preserved, and diagnostic vigilance maintained. The protocol also demonstrated potential to mitigate tourniquet‐induced hyperalgesia and postoperative inflammation, even during revision surgery, traditionally associated with a higher pain burden due to scar dissection and soft tissue reentry.

The incorporation of non‐pharmacologic adjuncts, such as TENS, in this case underscores the role of neuromodulation in enhancing postoperative analgesia without motor compromise. While TENS contributed to effective pain control in both surgical episodes, emerging evidence suggests that percutaneous PNS may offer complementary benefits by extending analgesia well into the subacute recovery phase. Although not utilized in this patient, PNS holds promise for future integration within opioid‐sparing protocols, particularly as a bridge following catheter discontinuation. Its application aligns with the principles of ERAS and may further reduce reliance on systemic analgesics.

While this single‐case design limits broad generalization, it reinforces the need for expanded multicenter studies that stratify by fracture pattern, surgical technique, and patient‐specific risk factors to minimize the risks associated with opioid dependence in orthopedic trauma. Future research should assess long‐term functional recovery, NPOU, and economic outcomes to guide the development of individualized, evidence‐based perioperative pain management strategies.

## Author Contributions

**Bi Mo:** conceptualization, data curation, formal analysis, visualization, writing – original draft, writing – review and editing. **Sandra Sacks:** data curation, formal analysis, writing – original draft, writing – review and editing. **Jerry Markar:** data curation, formal analysis, writing – original draft, writing – review and editing.

## Ethics Statement

The authors have nothing to report.

## Consent

Written informed consent was obtained from the patient for the publication of this case report and any accompanying images. A copy of the written consent is available for review upon request by the editor(s).

## Conflicts of Interest

The authors declare no conflicts of interest.

## Data Availability

Data and materials utilized to prepare for this case report were drawn from the patient's electronic medical chart.
